# Sensitivity Analysis of a New Cancer Risk Assessment Tool That Evaluates Salivary Polyamines

**DOI:** 10.7759/cureus.90217

**Published:** 2025-08-16

**Authors:** Motohiro Kurose, Masahiro Umeda, Sakiko Soutome, Teruyuki Niimi, Hideto Imura, Kayo Hayami, Masako Yoshimatsu, Takashi Ukai, Shuji Nomoto, Nagato Natsume

**Affiliations:** 1 Division of Research and Treatment for Oral and Maxillofacial Congenital Anomalies, School of Dentistry, Aichi Gakuin University, Nagoya, JPN; 2 Department of Oral Health, Nagasaki University Graduate School of Biomedical Sciences, Nagasaki, JPN; 3 Oral Management Center, Nagasaki University Hospital, Nagasaki, JPN; 4 Department of Surgery, Aichi Gakuin University Hospital, Nagoya, JPN

**Keywords:** cancer, pancreatic cancer, polyamine, preventive medicine, screening, sensitivity

## Abstract

Background: A method to assess cancer risk through salivary-polyamine analysis (SalivaChecker®, SalivaTech Co., Ltd., Tsuruoka, Japan) has been developed. However, studies evaluating the utility of this test in patients with cancer are lacking. Therefore, we evaluated the sensitivity of the SalivaChecker® test in patients with confirmed cancer.

Materials and methods: The SalivaChecker® test was performed in 66 patients with confirmed cancer. The risk of six types of cancer (lung, pancreatic, gastric, colorectal, breast (for women only), and oral cancers) was assessed.

Results: The overall cancer detection rate, which is defined as the rate of detecting the risk for any cancer in the individual, was 68.2%. The detection rates according to the cancer site were as follows: lung cancer (13/14), pancreatic cancer (8/14), gastric cancer (4/6), colorectal cancer (12/17), breast cancer (3/8), and oral cancer (5/7). The overall cancer site detection rate, which was defined as correctly identifying the cancer site, was 28.8%. The detection rates for individual cancers were as follows: lung cancer (4/14), pancreatic cancer (7/14), gastric cancer (3/6), colorectal cancer (1/17), breast cancer (3/8), and oral cancer (1/7).

Conclusion: The sensitivity of the SalivaChecker® was 68.2%. Future studies should assess the specificity of this test in patients without cancer to further evaluate its utility.

## Introduction

Malignant tumors are the leading cause of death among the Japanese population, with one in two people developing cancer and one in three dying from cancer [1]. If cancer is diagnosed and treated at an early stage, the prognosis is usually good. Various cancer screening tests are available; however, the screening rate in Japan is low owing to factors such as low awareness of preventive medicine, lack of coverage for preventive measures or screening under the universal health insurance system, and the time required for and invasiveness of cancer screening procedures [2]. Several tumor markers in the peripheral blood can be used to detect cancer; however, except for prostate-specific antigen for prostate cancer, they have low sensitivity for early-stage cancer and are not useful for screening [3]. In addition, some cancers, such as pancreatic cancer, are difficult to detect at an early stage [4].

In recent years, several methods have been developed to non-invasively and easily determine an individual’s risk of cancer, and some of these are used clinically. Currently, tests measuring microRNA in urine samples (miSignal®) [5], nematodes in urine samples (N-NOSE®) [6], or polyamines in saliva samples (SalivaChecker®) for determining the cancer risk are commercially available.

Human cells contain polyamine molecules with multiple primary amines within their structure. The intracellular concentration of polyamines is tightly regulated by controlling their biosynthesis, degradation, and transport in and out of the cell. Polyamines, which carry a positive charge, mainly bind to negatively charged RNA and promote nucleic acid and protein synthesis. Polyamines are essential for both cell division and proliferation [7]. In rapidly proliferating cells, such as cancer cells, polyamines are excessively produced within the cell, and polyamine levels in the saliva and urine are significantly increased in patients with cancer [8]. Moreover, several basic studies [9-19] have shown that the amount and type of salivary polyamines change in patients with cancer. Therefore, salivary polyamines are potential markers for early cancer diagnosis.

In this study, we focused on SalivaChecker® (SalivaTech Co., Ltd., Tsuruoka, Japan), a salivary polyamine-based test that is easy to administer in clinical dental practice. Although several preclinical studies have demonstrated that salivary polyamine levels are altered in cancer patients, no peer-reviewed clinical studies have assessed the diagnostic performance of SalivaChecker® in patients with confirmed cancer. For a screening tool to be clinically useful, high sensitivity and a low false-negative rate are essential. Therefore, in this preliminary study, we examined the sensitivity of SalivaChecker®, which is easy to perform in clinical dental practice, for cancer risk determination. This study was designed as an initial diagnostic investigation to evaluate the feasibility of using salivary polyamine analysis as a non-invasive cancer risk assessment tool in patients with confirmed malignancies.

Early detection of cancer significantly improves prognosis, but effective screening methods are limited for some cancers, such as pancreatic cancer. SalivaChecker® offers a non-invasive and easily accessible tool for assessing cancer risk through salivary polyamine analysis. Therefore, this preliminary study aims primarily to validate the sensitivity of SalivaChecker® in patients with confirmed malignancies as an initial step toward exploring its potential clinical utility in cancer screening.

## Materials and methods

Study design

This was a preliminary study, with patient registration at two facilities. The first patient was registered on December 13, 2023. The study protocol conformed to the ethical guidelines outlined in the Declaration of Helsinki and the Ethical Guidelines for Medical and Health Research Involving Human Subjects by the Ministry of Health, Labor, and Welfare of Japan. Ethical approval was obtained from the Institutional Review Board of Nagasaki University Hospital (approval number: 23082103). Written informed consent was obtained from all participants. The study protocol was registered with the University Hospital Medical Information Network Center on September 1, 2023 (approval number: UMIN000051986).

Participants

Patients with lung, pancreatic, colorectal, stomach, breast, or oral cancer who provided consent to participate in this study were included. All patients were admitted for surgery or chemotherapy for cancer and visited a dental clinic for preoperative oral care. Information regarding age, sex, cancer site, stage, histological type, and whether preoperative chemotherapy was administered was collected from the medical records.

Sample collection and analysis

After admission to the hospital, saliva samples were collected under the guidance of a dentist in the ward at the same time of day (7:30-8:00 am) on the morning of the day of surgery or chemotherapy. No patient had eaten since 9 pm the previous night or brushed their teeth that morning. We collected 0.1 mL of saliva in a special container. The samples were frozen and sent to SalivaTech Co., Ltd., where the amount and type of polyamines present were analyzed. An artificial intelligence tool determined the risk of developing six types of cancer: lung, pancreatic, colorectal, stomach, breast (for women only), and oral cancer. The SalivaChecker® is already commercially available and has been applied in clinical settings. We purchased the sample collection kit, collected our samples, and sent them to SalivaTech Co., Ltd. Later, we received the results by mail. The results were classified into four levels: A (very low risk), B (low risk), C (high risk), and D (very high risk), with C and D being judged as “at risk.”

The proportion of participants judged to be at risk for any of the six types of cancer (five types for men) was defined as the cancer detection rate. The proportion of correctly determined cancer sites was defined as the cancer site detection rate. We calculated the cancer and cancer site detection rates according to cancer type and examined the factors affecting these detection rates. For reference, data on 5,000 cases of SalivaChecker® test results in healthy subjects were obtained from Salivatech Co., Ltd., and compared with the results of the cancer patients in this study.

Statistical analysis

The target number of cases was set at 70. All statistical analyses were performed using SPSS Statistics version 26.0 (IBM Corp. Released 2019. IBM SPSS Statistics for Windows, Version 26.0. Armonk, NY: IBM Corp.). The relationship between categorical and continuous variables and the cancer risk rate was analyzed using Fisher’s exact test and the Mann-Whitney U test, respectively. Statistical significance was defined as a two-tailed p < 0.05.

## Results

Patient characteristics

After excluding two patients in whom obtaining saliva samples was difficult owing to xerostomia and two patients in whom postoperative pathological examination revealed no malignancy, 66 patients (35 men and 31 women) were included in this study. Participants’ ages ranged from 35 to 88 years (mean, 68.8 ± 10.9 years). Regarding the primary disease, 17, 14, 14, 8, 7, and 6 patients were diagnosed with colorectal, pancreatic, lung, breast, oral, and gastric cancer, respectively. Stage 1-2 and stage 3-4 cancers were diagnosed in 32 and 34 patients, respectively. Histologically, 50, 11, and 5 patients were diagnosed with adenocarcinoma, squamous cell carcinoma, and other types of cancer, respectively. Preoperative chemotherapy was administered to 17 patients (Table 1).

**Table 1:** Participant characteristics SD: standard deviation

Variable	Category	Number of patients (mean ± SD)
Sex	Male	35
	Female	31
Age	(Years)	68.8 ± 10.9
Primary disease	Colorectal cancer	17
	Pancreatic cancer	14
	Lung cancer	14
	Breast cancer	8
	Oral cancer	7
	Gastric cancer	6
Histology	Adenocarcinoma	50
	Squamous cell carcinoma	11
	Others	5
Stage	Stage 1-2	32
	Stage 3-4	34
Neoadjuvant chemotherapy	(-)	49
	(+)	17
Total		66

Cancer risk assessment

**Figure 1:**
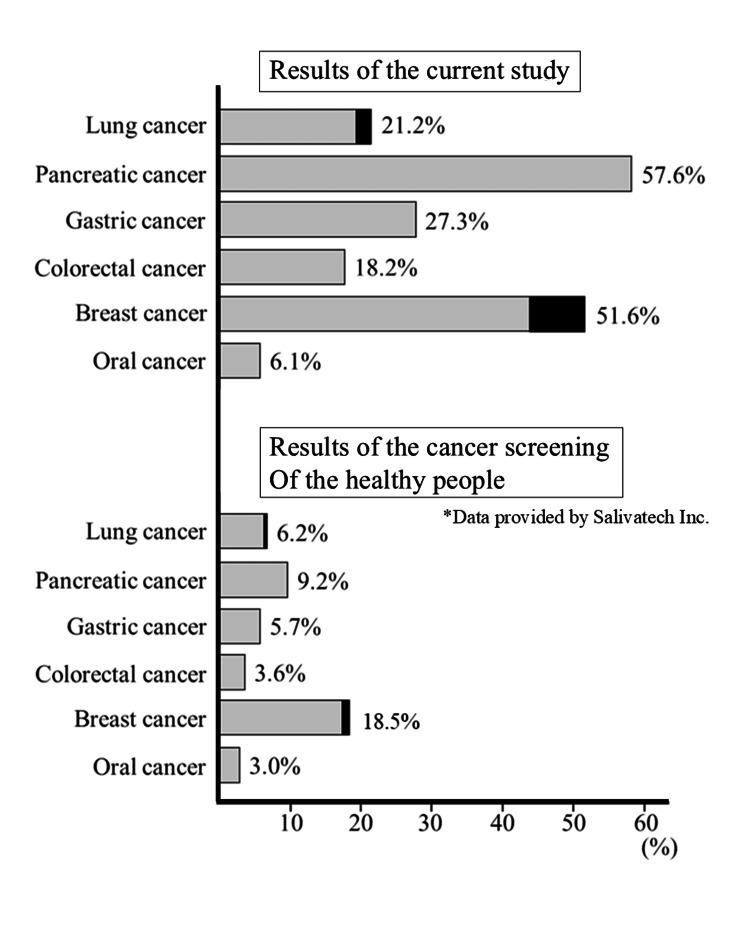
Cancer risk assessment Participants in this study exhibited a higher risk for cancer than health-checkup participants.

The results of the SalivaChecker® test indicated that 38, 18, 16, 12, and 4 patients were at risk (categories C or D) for pancreatic, stomach, lung, colorectal, and oral cancer, respectively. Furthermore, of the 31 women, 16 were at risk for breast cancer. In contrast, data from approximately 5,000 screening patients provided by SalivaTech (most of whom were presumed to be cancer-free) indicated that the risk of breast cancer was 18.5%, and the risk of other cancers ranged from 4% to 10% (the data of health examinees provided by the manufacturer). Thus, the risk rate in patients with cancer was higher in this study (Figure 1).

Cancer detection and site determination rates

The overall cancer detection rate was 45/66 (68.2%) patients. The cancer detection rates according to site were as follows: lung cancer, 13/14 (92.9%) patients; oral cancer, 5/7 (71.4%) patients; colorectal cancer, 12/17 (70.6%) patients; gastric cancer, 4/6 (66.7%) patients; pancreatic cancer, 8/14 (57.1%) patients; and breast cancer, 3/8 (37.5%) patients. Except that for breast cancer, these rates were relatively high.

**Figure 2:**
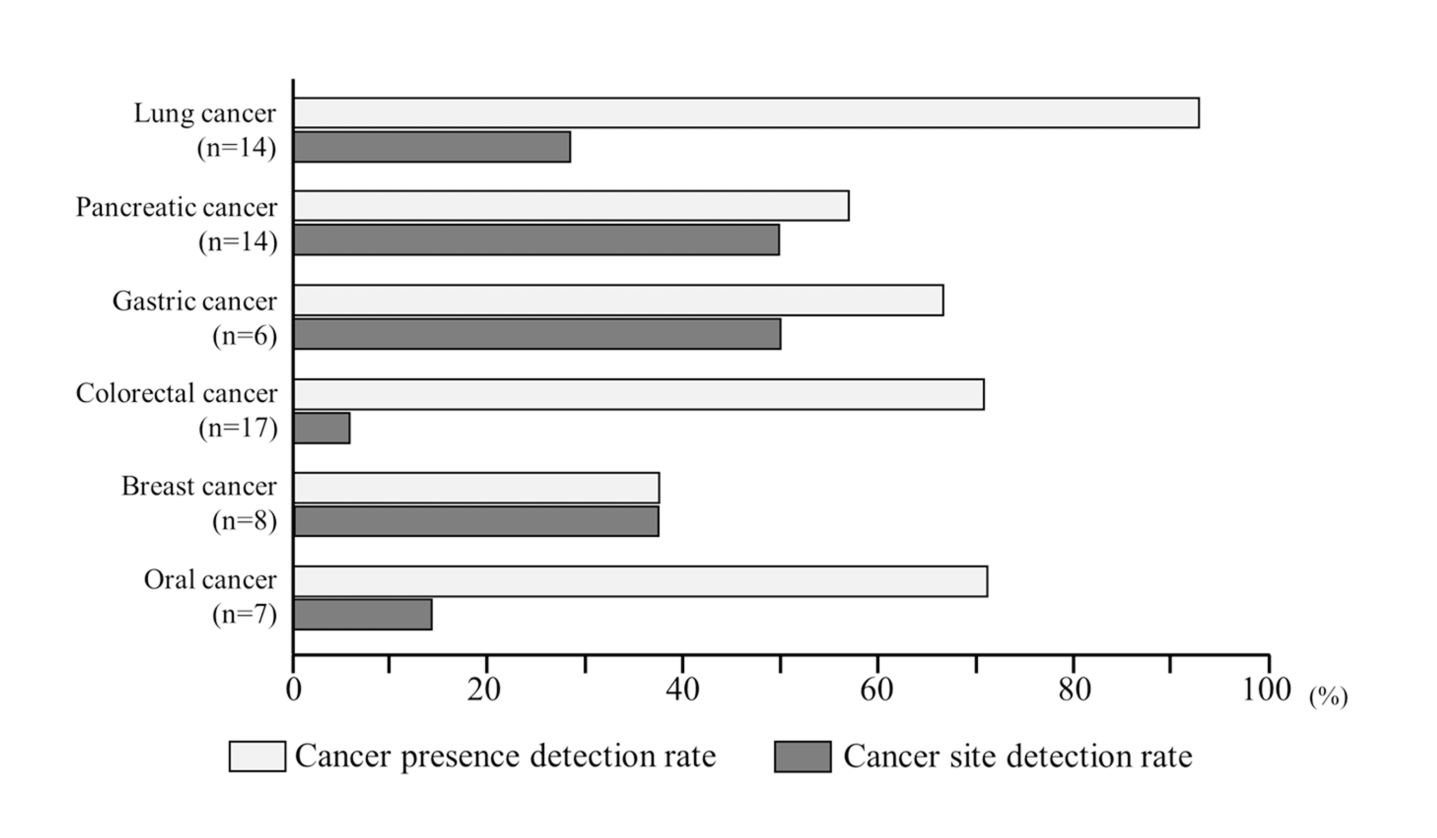
Cancer and cancer site detection rates A significant difference between the cancer and cancer site detection rates is observed for lung, colorectal, and oral cancers; however, the difference between the two is small for pancreatic, gastric, and breast cancers.

The overall cancer site detection rate was 19/66 (28.8%) patients. According to the cancer site, the rates were as follows: pancreatic cancer, 7/14 (50%) patients; gastric cancer, 3/6 (50%) patients; breast cancer, 3/8 (37.5%) patients; lung cancer, 4/14 (28.6%) patients; oral cancer, 1/7 (14.3%) patients; and colorectal cancer, 1/17 (5.9%) patients. Although a higher number of patients with lung and colorectal cancers were determined to have a cancer risk at any site, the site-detection rate was low. However, 8 of 14 (57%) of patients with pancreatic cancer were determined to have cancer risk, and the majority of these were correctly identified as being at risk for pancreatic cancer (Figure 2).

Factors affecting the accuracy of cancer and cancer site detection

Univariate analysis revealed that the cancer detection rate was significantly higher in older adults; however, no significant correlation was found between age and site-detection rate. The cancer detection rate (26/34 (76.5%) vs. 19/32 (59.4%)) and site-detection rate (13/34 (38.2%) vs. 6/32 (18.8%)) tended to be higher in patients with stage 3-4 cancer than in patients with stage 1-2 cancer; however, the difference was not statistically significant. According to the cancer site, the cancer detection rate was significantly higher in patients with lung cancer than in those with other cancer types (13/14 (92.9%) vs. 32/52 (61.5%), p=0.027). In contrast, the cancer detection rate was significantly lower in patients with colorectal cancer than in those with other cancer types (1/17 (5.9%) vs. 18/49 (36.7%), p=0.015). Additionally, no correlation was found between the cancer cancer site-detection rates and sex, histological type, and the administration of preoperative chemotherapy (Table 2).

**Table 2:** Factors associated with determining the presence of cancer and the location of cancer

Variable	Category	Number of patients	Presence of cancer		x^2^ value	p-value	Site of cancer		x^2^ value	p-value
			Correct	Incorrect			Correct	Incorrect		
Sex	Male	35	25	10	0.362	0.603	12	23	1.099	0.415
	Female	31	20	11	-	-	7	24	-	-
Age	<70 years	26	13	13	6.537	0.015	5	21	1.911	0.266
	≥70 years	40	32	8	-	-	14	26	-	-
Primary disease	Colorectal cancer	17	12	5	8.273	0.142	1	16	9.755	0.082
	Pancreatic cancer	14	13	1	-	-	4	10	-	-
	Lung cancer	14	8	6	-	-	7	7	-	-
	Breast cancer	8	3	5	-	-	3	5	-	-
	Oral cancer	7	5	2	-	-	1	6	-	-
	Gastric cancer	6	4	2	-	-	3	3	-	-
Histology	Adenocarcinoma	50	35	15	0.719	0.698	16	34	0.798	0.671
	Squamous cell carcinoma	11	8	3	-	-	2	9	-	-
	Others	5	2	3	-	-	1	4	-	-
Stage	Stage 1-2	32	19	13	1.746	0.29	6	26	1.848	0.277
	Stage 3-4	34	26	8	-	-	13	21	-	-
Neoadjuvant chemotherapy	(-)	49	34	15	0.094	0.77	12	37	1.588	0.228
	(+)	17	11	6	-	-	7	10	-	-
Total		66	45	21	-	-	19	47	-	-

## Discussion

This study represents a preliminary diagnostic investigation aimed at exploring the clinical utility of the SalivaChecker® test in identifying cancer risk among patients with confirmed malignancy. While the findings are promising, they should be interpreted with caution given the exploratory nature of the research.

Since 1980, malignant tumors have been the leading cause of death in Japan, and the number of patients and deaths has increased annually. Data for the number of deaths according to cancer site show that for men, the most common types are lung, stomach, colorectal, and pancreatic cancers; in contrast, colorectal, lung, pancreatic, and breast cancers are the most common in women [20]. Early detection and treatment are crucial to improve cancer prognosis. Among the various cancer screening methods currently available, effective tests include fecal occult blood testing and colonoscopy for colorectal cancer [21], upper gastrointestinal radiography and gastroscopy for gastric cancer [22], radiography and computed tomography for lung cancer [23], and mammography for breast cancer [24]. In contrast, tumor markers used for cancer diagnosis through blood tests, with a few exceptions, generally do not have high sensitivity for early-stage cancers and are not effective for early cancer diagnosis [25-40]. Tumor markers are commonly used to monitor treatment effectiveness and diagnose recurrence. Among malignant tumors, pancreatic cancer has the worst prognosis and is challenging to diagnose early using imaging or tumor markers. The sensitivities of the tumor markers used for the six types of cancer examined in this study range from 20% to 80% (Table 3) [25-40]. However, the sensitivity of all markers is low for early-stage cancer, and it increases after cancer has progressed, such as when distant metastasis occurs.

**Table 3:** Sensitivity of tumor markers clinically applied to the six types of cancer CEA: carcinoembryonic antigen, NSE: neuron-specific enolase, ProGRP: pro-gastrin-releasing peptide, CYFRA21-1: cytokeratin-19 fragment, SCC antigen: squamous cell carcinoma antigen, CA: cancer antigen, Span-1: S-pancreas-1 antigen, Dupan-2: Duke pancreatic monoclonal antigen type 2, TPA: tissue polypeptide antigen, AFR: albumin-to-fibrinogen ratio

Author, year	Site of cancer (histology)	Tumor marker	Sensitivity
Wu et al., 2018 [25]	Ling cancer	CEA	59.0%
Lyubimova et al., 2022 [26]	Lung cancer (small cell carcinoma)	NSE	64.1%
Lyubimova et al., 2022 [26]	Lung cancer (small cell carcinoma)	ProGRP	71.0%
Zhao et al., 2014 [27], Ma et al., 2015 [28]	Lung cancer (squamous cell carcinoma)	CYFRA21-1	59.7-77.1%
Sánchez et al., 1994 [29]	Lung cancer (squamous cell carcinoma)	SCC antigen	47.7%
Ma et al., 2015 [28]	Lung cancer (adenocarcinoma)	CA125	44.9%
Haglund et al., 1986 [30]	Pancreatic cancer	CA19-9	78.0%
Kitayama et al., 1990 [31]	Pancreatic cancer	Span-1	81.3%
Kitayama et al., 1990 [31]	Pancreatic cancer	Dupan-2	66.7%
Haglund et al., 1986 [30]	Pancreatic cancer	CEA	54.0%
Kawa et al., 1990 [32]	Pancreatic cancer	CA50	73.0%
Haglund et al., 1986 [30]	Pancreatic cancer	CA125	45.0%
Huang et al., 2021 [33]	Colorectal cancer	CEA	65.8%
Huang et al., 2021 [33]	Colorectal cancer	CA19-9	42.3%
Plebani et al., 1996 [34]	Colorectal cancer	TPA	67.0%
Persson et al., 1988 [35]	Colorectal cancer	CA50	47.0%
Yu et al., 2016 [23]	Gastric cancer	CA19-9	19.0%
Yu et al., 2016 [36]	Gastric cancer	CEA	22.7%
Li et al., 2022 [37]	Gastric cancer	CA125	19.8%
Li et al., 2022 [37]	Gastric cancer	AFR	16.0%
Cheung et al., 2000 [38]	Breast cancer	CEA	46.0-53.0%
Cheung et al., 2000 [38]	Breast cancer	CA15-3	54.0-87.0%
Gion et al., 2001 [39]	Breast cancer	CA27-29	87.0%
Yoshimura et al., 1988 [40]	Oral cancer	SCC antigen	43.4%

The requirements for a cancer screening test include, first and foremost, high sensitivity, meaning a low false-negative rate, and secondly, high specificity. This study targeted patients diagnosed with cancer and aimed to evaluate the sensitivity of this test. The cancer detection rate was 68.2%, which was comparable to or higher than that of many conventional blood-based tumor markers. Considering that specimen collection is easy and can be performed at home, this test is considered useful. In contrast, although the SalivaChecker® test can identify the risk for six types of cancer, the actual cancer site detection rate was 28.6%, which is not satisfactory. However, the difference between the cancer and cancer site detection rates was small for pancreatic, gastric, and breast cancers, suggesting that the presence of these three types of cancer can be determined relatively accurately. Although highly accurate cancer screening using endoscopy and mammography is possible for stomach and breast cancer, respectively, few useful cancer screening methods are available for pancreatic cancer.

This study evaluated the sensitivity of the test; however, in the future, it will be necessary to assess its specificity in individuals without cancer. According to the data provided by the manufacturer, the rate of identification of the six cancer types during cancer screening ranged from 3% to 18%, which is lower than the positivity rate observed in patients with cancer in this study. This finding suggests that the test may be useful from the perspective of specificity. However, data on whether individuals categorized as having a risk for cancer using the SalivaChecker® test were subsequently diagnosed with cancer using detailed cancer screening are lacking. Therefore, we are planning a clinical study to determine the specificity of the SalivaChecker® test in patients classified as cancer-free based on the results of screening tests.

Furthermore, as this study was conducted using data from a limited number of institutions, external validation using independent cohorts from other institutions is essential to confirm the generalizability and clinical applicability of the SalivaChecker® test. Although this preliminary study demonstrated promising sensitivity, external validation using independent cohorts from multiple institutions is essential to confirm the generalizability and clinical applicability of SalivaChecker®. Future studies should focus on larger, diverse populations to establish robust evidence for its use as a reliable cancer screening tool.

This study has several limitations, including a small and unevenly distributed sample across cancer types, a lack of direct specificity evaluation, and the absence of data on potential confounding factors such as comorbidities and concurrent treatments. Future studies should address these issues using larger, more diverse cohorts and collecting comprehensive clinical background data to validate the reproducibility and clinical utility of the SalivaChecker® test.

## Conclusions

This preliminary study demonstrated that salivary polyamine analysis using the SalivaChecker® test may offer a non-invasive method for assessing cancer risk, with an overall detection rate comparable to conventional tumor markers. While the test showed promise, particularly for cancers that are difficult to detect early, such as pancreatic cancer, its ability to identify the specific cancer site remains limited. These findings support the potential utility of this method in cancer screening, particularly in settings where conventional screening is challenging, and warrant further investigation in larger, population-based studies.
